# Circulating Tumor DNA Profiling Defines Risk Classification in Patients With Ewing Sarcoma: A Report From the Children’s Oncology Group and the LEOPARD Study

**DOI:** 10.1200/JCO-26-00285

**Published:** 2026-09-15

**Authors:** David S. Shulman, Kelly Klega, Nan Chen, Allen Buxton, Elliot Gohn, Sarah Sexton, Catherine Clinton, Mohammad Tanhaemami, Carrie Cibulskis, Edwin Choy, Thomas Cash, Kris Ann P. Schultz, Leo Mascarenhas, Rochelle Bagatell, Christopher Kuo, Brian K. Turpin, Bhuvana A. Setty, Bradley D. DeNardo, Douglas S. Hawkins, Michael W. Bishop, Avanthi Tayi Shah, Luke D. Maese, Julia L. Glade Bender, Damon Reed, Mark D. Krailo, Natalie DelRocco, Wendy B. London, Katherine A. Janeway, Steven G. DuBois, Brian D. Crompton

**Affiliations:** 1 Dana-Farber/Boston Children’s Cancer and Blood Disorders Center, Harvard Medical School, Boston, MA; 2 Broad Institute of Harvard and MIT, Cambridge, MA; 3 Children’s Oncology Group, Monrovia, CA; 4 Department of Population and Public Health Sciences, Keck School of Medicine, University of Southern California, Los Angeles, CA; 5 Mass General Brigham Cancer Institute, Harvard Medical School, Boston, MA; 6 Emory University School of Medicine, Aflac Cancer and Blood Disorders Center at Children’s Healthcare of Atlanta, Atlanta, GA; 7 Cancer and Blood Disorders, Children’s Minnesota, Minneapolis, MN; 8 Guerin Children’s and Cedars-Sinai Cancer, Cedars Sinai Health Sciences University, Los Angeles, CA; 9 Children’s Hospital of Philadelphia and University of Pennsylvania Perelman School of Medicine, Philadelphia, PA; 10 Cancer and Blood Disease Institute, Children’s Hospital Los Angeles, Los Angeles, CA; 11 Keck School of Medicine, University of Southern California, Los Angeles, CA; 12 Cincinnati Children’s Hospital Medical Center, Cincinnati, OH; 13 Nationwide Children’s Hospital, The Ohio State University, Columbus, OH; 14 Brown University and Hasbro Children’s Hospital, Providence, RI; 15 Seattle Children’s Hospital and University of Washington, Seattle, WA; 16 St Jude Children’s Research Hospital, Memphis, TN, USA; 17 Arkansas Children’s Hospital, Little Rock, AR; 18 University of Texas Southwestern Medical Center, Dallas, TX; 19 University of Utah, Huntsman Cancer Institute, and Primary Children’s Hospital, Salt Lake City, UT; 20 Memorial Sloan Kettering Cancer Center, New York, NY

## Abstract

**PURPOSE:**

Identification of discrete risk groups remains a high priority for patients with Ewing sarcoma (EWS). We sought to prospectively validate circulating tumor DNA (ctDNA) as a prognostic factor and develop clinical-molecular risk groups.

**METHODS:**

We conducted a prospective investigator-initiated biology study for patients with localized EWS (LEOPARD) and embedded ctDNA analysis into the North American frontline metastatic study AEWS1221. Eligible patients were younger than 50 years with newly diagnosed EWS. All patients provided a baseline blood sample for analysis, which was subjected to ultralow-pass whole-genome sequencing and hybrid capture panel sequencing for ctDNA quantification, fusion detection, and characterization of *STAG*2 and *TP*53 alterations. Serial ctDNA sequencing was conducted on a subset of patients in each study. We tested for associations between ctDNA burden and secondary genomic events, and clinical features and outcomes.

**RESULTS:**

One hundred forty patients with localized disease and 255 with metastatic disease provided evaluable pretreatment samples for ctDNA analysis. Elevated baseline ctDNA was associated with stage, tumor size, primary site, indeterminate pulmonary nodules, and metastatic pattern. Elevated pretreatment ctDNA burden was associated with inferior outcomes in patients with localized (n = 140, hazard ratio [HR] = 2.36, *P* = .032) and metastatic disease (n = 255, HR = 2.15, *P* = .001). Patients with metastatic disease and *TP*53 variants and/or persistent on-therapy ctDNA had dismal outcomes. Patients with localized disease, low ctDNA, small tumors, and favorable genomics had no events and constitute a novel low-risk group. Among patients with metastatic disease, those with lung-only disease, low ctDNA, and favorable genomics represent an intermediate-risk group.

**CONCLUSION:**

This study prospectively validates pretreatment ctDNA burden as prognostic in EWS. Risk groups that integrate ctDNA burden with clinical-molecular features differentiate patients with low-, intermediate-, and high-risk disease.

## INTRODUCTION

Ewing sarcoma (EWS) is an aggressive, fusion-positive sarcoma that primarily affects children and young adults.^1^ The presence of detectable metastatic disease at diagnosis is currently the strongest adverse prognostic factor. Patients with localized versus metastatic disease have a 5-year event-free survival (EFS) of nearly 80%^2^ versus <40%.^1^ Despite these disparate outcomes, most patients receive five-drug interval-compressed chemotherapy and local control interventions.^2–4^ The long-term burden of treatment-related toxicity is high.^5,6^ Validated, biologically informed prognostic factors are needed to inform risk-stratified therapy.

EWS is characterized by FET-ETS fusions (most commonly *EWSR1::FLI1*) that define the disease and secondary deleterious mutations in *TP*53 and *STAG*2 in a minority of tumors.^7–11^ A recent analysis of patients with localized EWS confirmed that patients with *STAG*2 mutations have a high risk of relapse.^12^ In parallel, our group and others have shown in retrospective analyses that patients with elevated pretreatment circulating tumor DNA (ctDNA) have inferior outcomes.^13–15^ The prognostic utility of ctDNA in the context of other factors has not previously been prospectively studied.

We conducted the LEOPARD study (liquid biopsy in Ewing sarcoma and osteosarcoma as a prognostic and response diagnostic) to prospectively study ctDNA in localized EWS (ClinicalTrials.gov identifier: NCT06068075). In parallel, we embedded evaluation of ctDNA as an exploratory analysis in a Children’s Oncology Group (COG) phase III trial for patients with metastatic EWS (AEWS1221).^16^ We present validation of the prognostic impact of pretreatment ctDNA burden and integration of clinical-molecular features to define risk groups fit for incorporation into future trials.

## METHODS

### Study Design and Participants

#### Localized EWS (LEOPARD)

The LEOPARD study was a prospective, multicenter, biology study. Patients enrolled through one of 12 study centers or the COG study, Project:EveryChild (ABTR18B1-Q, Fig 1A). All patients or their legal guardians provided informed consent with assent as appropriate. The LEOPARD study was approved by each Institutional Review Board (IRB).

Patients age 1-50 years with newly diagnosed, nonresected, localized EWS enrolled. All patients were treated starting in 2017 in North America where interval-compressed vincristine/doxorubicin/cyclophosphamide alternating with ifosfamide/etoposide (VDC/IE) was the standard of care. For patients enrolled through study centers, blood was obtained at baseline and throughout treatment (Data Supplement, Fig S1A, online only). For patients enrolled through Project: EveryChild, only a baseline blood sample was obtained. Some time points could not be obtained during the COVID-19 pandemic.

#### Metastatic EWS (AEWS1221)

AEWS1221 was a prospective randomized phase III trial of interval-compressed VDC/IE with or without ganitumab for patients younger than 50 years with newly diagnosed metastatic EWS (ClinicalTrials.gov identifier: NCT02306161).^16^ As published, there was no evidence of difference in outcomes across the two arms, and thus, our study population was treated as a single cohort. Blood collection for ctDNA analysis was included as an optional study. Initially, the trial included only baseline ctDNA. Subsequent time points were added during study amendments (Data Supplement, Fig S1B).

The National Cancer Institute’s (NCI) Pediatric Central IRB approved AEWS1221. All sites either used the central IRB or obtained local IRB approval. All patients or their legal guardians provided written informed consent.

### Circulating Tumor DNA and Tissue Analysis

ctDNA analysis was conducted uniformly in a single laboratory across both studies as previously described (Data Supplement, Methods).^17^

### Statistical Considerations

Independent and dependent variables are defined in the Data Supplement, Methods. The LEOPARD study was designed to enroll a minimum of 125 patients to provide 80% power to detect a 20% difference in 2-year EFS: 60% for patients with ≥0.5% ctDNA versus 80% for patients with <0.5% ctDNA at baseline (one-sided log-rank test; α = .05). The sample size of the AEWS1221 cohort was driven by the trial’s primary objective for treatment comparison.^16^

Survival analyses landmarked from a given time point included only patients with an evaluable sample at that time point. Kaplan-Meier methods were used to generate EFS and overall survival (OS) curves and to calculate point estimates with standard errors according to the Greenwood method.^18^ Tests for equality of survival curves by patient characteristics were conducted using a one-sided log-rank test for LEOPARD and two-sided log-rank test for AEWS1221. After verifying the proportional hazards (PH) assumption, univariate and multivariable Cox PH regression models were used to assess the prognostic effect of ctDNA and to estimate hazard ratios (HRs) for EFS and OS, stratified by ctDNA level. Multivariable models controlled for known prognostic variables in EWS. Covariate inclusion in multivariable models was defined by clinical investigators based on disease expertise before analysis of outcome data. Models were also fit with linear functional forms for covariates (age, baseline ctDNA burden).

A one-sided *P* value was considered significant as prespecified in the LEOPARD protocol. A two-sided *P* value of ≤.05 was considered significant in AEWS1221 analyses. All statistical analyses were performed using Stata 19 (RRID: SCR_012763), R Statistical Software Version 4.5.0 (RRID: SCR_001905), or SAS Version 9.4 (RRID:SCR_008567).

## RESULTS

### Patient Characteristics

From October 2017 to October 2023, 143 patients with localized EWS met initial eligibility criteria and were included in the LEOPARD study, either at collaborating primary sites (n = 53 patients) or via submission of a baseline plasma sample to the COG Project:EveryChild study (ABTR18B1-Q; n = 90 patients; Fig 1A). Two patients were found to be ineligible, including one patient who was found to have a non-EWS diagnosis and one patient found to have metastatic disease. One patient had poor sequencing quality of their baseline plasma sample and was excluded. The LEOPARD cohort therefore included 140 patients with evaluable pretreatment ctDNA measurements (Table 1 and Data Supplement, Table). The median age at enrollment was 14.3 years (range: 2.2-35.5), and 65% was male. These patients provided 407 on-treatment and relapse samples (Data Supplement, Fig S1A). The median follow-up time for the 102 patients without an EFS event was 3.8 years (range, 0.4-7.0).

AEWS1221 enrolled 298 patients from December 2014 to March 2019. Of these, 273 consented to provide blood samples for ctDNA analysis (Fig 1B). We found two patients with non–FET-ETS family fusions detected by ctDNA (*CIC::DUX*4 and *EWSR1::WT1*), highlighting the potential diagnostic utility of this approach. After excluding these patients and seven patients without any evaluable samples, the AEWS1221 cohort included 264 patients (Table 1). The median age at enrollment was 15.0 years (range: 0.0-38.0), and 56% was male. These patients provided 684 serial samples for analysis (Data Supplement, Fig S1B). Eight patients were excluded from analysis because of elevated baseline ctDNA ≥5% by ultralow-pass whole-genome sequencing (ULP-WGS), but for whom no fusion was identified by TranSS-Seq or in the clinical case report forms. There was one patient with no baseline sample. The primary cohort for baseline survival analyses therefore included 255 patients. The median follow-up time for patients without an EFS event was 4.96 years (range: 0.01-7.99).

### Landscape of ctDNA Burden, *STAG*2 Mutations, and *TP*53 Mutations in Localized EWS

Among 140 patients with localized EWS, 64% (n = 90 of 140) had fusion-based detectable ctDNA burden at baseline (median baseline ctDNA burden: 3%; range: undetectable, 77%; Figs 2A and 2C).

Diagnostic tissue was available for 82 of 140 patients on the LEOPARD study. Tissue-based sequencing identified an additional 27 fusions in patients with undetectable baseline ctDNA burden. Fusions were identified in baseline ctDNA or tissue in 84% (117 of 140) of patients. The distribution of fusions was typical of EWS^1^ with 92% (107 of 117) of patients with *EWSR1::FLI1*, 8% (9 of 117) with *EWSR1::ERG*, and one with *FUS::ERG*. Among 119 patients with baseline ctDNA ≥0.5% and/or tumor tissue, *STAG*2 alterations were detected in 8 (7%) and *TP*53 mutations in 8 (7%). One patient had concurrent *STAG*2 and *TP*53 mutations at baseline.

### Landscape of ctDNA Burden, *STAG*2 Mutations, and *TP*53 Mutations in Metastatic EWS

Among patients with metastatic disease, 94% (239 of 255) of patients had detectable ctDNA at baseline (median baseline ctDNA burden: 26.6%; range: undetectable, 100%; Figs 2B and 2D). A total of 92% (234 of 255) of patients had ≥0.5% ctDNA at baseline. By ULP-WGS, the median baseline ctDNA burden was 17% (range: undetectable, 88%). In one instance, we found a patient with a known FET-ETS fusion for whom ctDNA was elevated when assessed by ULP-WGS (27%), but negative by TranSS-Seq–based fusion detection. To ensure consistency across the cohort in the primary analysis, this patient was categorized as having undetectable baseline ctDNA.

Among the 255 patients with baseline samples, the distribution of fusions was typical of EWS,^1^ with 84% (214 of 255) *EWSR1::FLI1*, 7.5% (19 of 255) *EWSR1::ERG*, 0.8% (2 of 255) *EWSR1::FEV*, 0.8% (2 of 255) *EWSR1::ETV1*, and 0.8% (2 of 255) *FUS::ERG*. Sixteen patients with a baseline sample did not have a fusion identified at any time point.

Among the 234 patients with metastatic disease and detectable ctDNA burden ≥0.5%, *STAG*2 mutations were detected in 11% (26 of 234) and *TP*53 mutations were detected in 6.4% (15 of 234). Four patients had concurrent *TP*53 and *STAG*2 mutations at baseline. Among the 31 patients with relapse samples, 17 patients had detectable ctDNA including four patients with an *STAG*2 mutation and three with a *TP*53 mutation. In three of these cases, the mutations were not detected at baseline (Data Supplement, Fig S2).

### Clinical Features Are Associated With ctDNA Burden at Diagnosis

Patients with metastatic disease had significantly higher median ctDNA burden at diagnosis compared with those with localized disease (Table 2, Data Supplement, Fig S3; *P* < .001). Within each study population, we separately tested for associations between baseline ctDNA detection and baseline clinical features (Table 2). Large tumors, indeterminate pulmonary nodules, and pelvic bone primary were significantly associated with elevated ctDNA burden in localized EWS (*P* = .032, *P* = .018, and *P* = .001, respectively). Age was not associated with ctDNA burden among patients with localized EWS. Age ≥14 years, pelvic bone primary, and extrapulmonary metastatic disease were associated with higher ctDNA burden in metastatic EWS (*P* = .002, *P* <.001, and *P* < .001, respectively).

### Pretreatment ctDNA Burden Is Prognostic in EWS

In the primary preplanned analysis of patients with localized EWS, ctDNA burden ≥0.5% was significantly associated with inferior EFS (2-year EFS, 79% [±SE 4.4%]) compared with ctDNA burden <0.5% (91% [±SE 4.2%], *P* = .014, Fig 3A) and OS (2-year OS, 93% [±SE 2.8%] *v* 100% [±SE 0%], *P* = .015, Fig 3B). An additional preplanned secondary cut point of 3% ctDNA burden was evaluated.^13^ Patients with ≥3% ctDNA burden had an inferior EFS compared with patients with <3% ctDNA burden (2-year EFS, 79% [±SE 5.0%] *v* 87% [±SE 4.2%], *P* = .053, Data Supplement, Fig S4A) and OS (2-year OS, 94% [±SE 3.0%] *v* 97% [±SE 2.2%], *P* = .037, Data Supplement, Fig S4B). Within patients with localized disease, an analysis of ctDNA as a continuous variable did not identify a significant association with EFS (*P* = .213). Among the 38 EFS events on LEOPARD, there were four patients with second malignancy and one with death as the first event.

Mirroring our prior analysis,^13^ patients with metastatic EWS and ctDNA burden ≥0.5% versus <0.5% had nominally lower EFS point estimates (2-year EFS, 41% [±SE 3.3%]; *v* 68% [±SE 10.8%], *P* = .055, Data Supplement, Fig S4C) and OS point estimates (2-year OS, 73% [±SE 3.0%] *v* 84% [±SE 8.7%], *P* = .218, Data Supplement, Fig S4D). Given that <10% of patients with metastatic disease had ctDNA <0.5%, we evaluated a 10-fold higher cut point and ctDNA as a continuous variable. Patients with a ctDNA burden ≥5% had an inferior 2-year EFS (38% [±SE 3.4%] *v* 71% [±SE 7.0%], *P* <.001, Fig 3C) and OS (70% [±SE 3.2%] *v* 88% [±SE 5.1%], *P* = .006, Fig 3D) compared with patients with <5% ctDNA at baseline. Evaluating ctDNA as a continuous predictor, the HR for the EFS event for each ten-percentage point increase in ctDNA burden was 1.16 (95% CI, 1.09 to 1.23, *P* < .001). Among the 157 EFS events on AEWS1221, there were nine patients with a second malignancy and five with death as the first event.

### Residual On-Treatment ctDNA Identifies Patients With Metastatic EWS at High Risk of Relapse

Longitudinal ctDNA samples were available for 51 patients with localized EWS. Only six patients had >0.5% ctDNA at any time point from precycle 2 and beyond, excluding relapse. We tested for an association between precycle 2 ctDNA burden (detectable *v* undetectable) and did not find a significant difference in EFS or OS (n = 33, *P* = .258 and *P* = .374, respectively, Data Supplement, Figs S5A–S5B) in this small cohort. There were too few patients with elevated ctDNA burden at subsequent time points for statistical analyses.

Among patients on AEWS1221, precycle 2 ctDNA collection was added to the protocol with an amendment, and samples were therefore only available for 35 patients. Within this group, patients with ≥0.5% ctDNA had dismal EFS that was significantly inferior to patients with <0.5% ctDNA (2-year EFS, 5.3% [±SE 5.1%] *v* 63% [±SE 12%], *P* < .001, Fig 4A). This group of patients had inferior OS as well (2-year OS, 42% [±SE 11%] *v* 81% [±SE 9.8%], *P* = .004, Fig 4B). Precycle 3, elevated ctDNA burden ≥0.5% was not associated with an inferior EFS (*P* = .104, Data Supplement, Fig S5C) but was associated with OS (*P* = .016, Data Supplement, Fig S5D). We evaluated patients with end-of-consolidation samples descriptively and show that 11 of 13 patients with detectable end-of-consolidation ctDNA had disease relapse/progression at that time (Data Supplement, Fig S6). One patient with elevated end-of-consolidation ctDNA relapsed shortly thereafter, and one did not relapse after extended followup. Two patients with relapse at the end of consolidation had negative ctDNA testing.

### Baseline *STAG*2 and *TP*53 Alterations Are Associated With Survival

We next tested for associations between baseline detection of *STAG*2 and *TP*53 mutations and EFS in patients with localized disease and available tissue or a baseline plasma sample with ctDNA burden ≥0.5% (n = 119). Patients with *STAG*2*^mut^* disease had similar outcomes compared with patients with *STAG*2*^WT^* disease (2-year EFS, 100% [±SE 0%] *v* 80% [±SE 3.9%], *P* = .364, Data Supplement, Fig S5E). Patients with *TP*53*^mut^* disease had inferior outcomes compared with patients with *TP*53*^WT^* disease (2-year EFS, 67% [±SE 19%] *v* 82% [±SE 3.7%], *P* = .009, Data Supplement, Fig S5F).

For patients with metastatic disease, *STAG*2*^mut^* disease detectable in baseline ctDNA was associated with inferior EFS compared with patients with *STAG*2*^WT^* disease (2-year EFS, 15% [±SE 7.1%] *v* 44% [±SE 3.5%], *P* = .002, Fig 4C). Patients with *TP*53*^mut^* detectable in ctDNA universally experienced events within 2 years of enrollment, compared with a 2-year EFS of 44% (±SE 3.4%) for patients with *TP*53*^WT^* disease (*P* < .001, Fig 4D).

### Integration of Clinical Features, Molecular Alterations, and Pretreatment ctDNA Burden Identifies Discrete Prognostic Risk Groups

Complete data were available for 134 patients with localized disease to perform a multivariable analysis. In a model that included baseline ctDNA burden (≥/<0.5%), tumor size, and age, elevated ctDNA had a HR of 2.3 (95% CI, 0.99 to 5.32; *P* = .053; Fig 5A).

Multiple recent studies have demonstrated that patients with large tumors and those with *STAG*2 and *TP*53 mutations have inferior outcomes.^10–12,19^ We compared outcomes between patients with <0.5% baseline ctDNA burden, <8cm tumors, AND *STAG*2*^WT^* and *TP*53*^WT^* disease (low-risk group) and all other patients (intermediate-risk group; Fig 5B). Patients in the low-risk group had outstanding outcomes compared with those in the intermediate-risk group (2-year EFS, 100% [±SE 0%] *v* 81% [±SE 3.6%], *P* = .0077, Fig 5C). These low-risk patients had superior OS as well, with no deaths observed through end of follow-up (*P* = .0366, Fig 5D).

Among patients with metastatic disease, we evaluated baseline ctDNA burden ≥5% in a multivariable model. After controlling for age, primary site, and metastatic pattern, ctDNA and metastatic pattern remained prognostic (Fig 5E). Given that ctDNA detection was required to identify *STAG*2 and *TP*53 mutations, we did not include these variables in the multivariable model.

To identify discrete risk groups among patients with metastatic disease, we compared outcomes for patients with hypothesized intermediate-risk disease (<5% ctDNA, lung-only disease, and *STAG*2*^WT^*/*TP*53*^WT^* disease) versus high-risk disease (all other patients; Fig 5F). We found that patients with intermediate-risk metastatic disease had superior 2-year EFS and OS (83% [±SE 8.8%] *v* 40% [±SE 3.2%]; *P* = .006; and 100% [±SE 0%] *v* 71% [±SE 3.0%], *P* = .019, respectively; Figs 5G and 5H) compared with high-risk metastatic patients.

## DISCUSSION

In prospective cohorts of patients with localized and metastatic EWS, we validate that pretreatment ctDNA burden is prognostic. In localized EWS, we define a group with small tumors, low ctDNA, and favorable genomics who have excellent outcomes. Similarly, in metastatic disease, we identify patients with lung-only disease, low ctDNA, and favorable genomics who have outcomes in the range of those typically seen in patients with localized disease. To our knowledge, this analysis is the first to prospectively validate pretreatment ctDNA in patients receiving contemporary therapy and define discrete clinical-molecular risk groups broadly applicable to patients with EWS.

Multiple prior studies have suggested that ctDNA burden at diagnosis is prognostic among patients with EWS.^13–15^ In localized EWS, we found that elevated ctDNA burden was associated with larger tumor size, pelvic bone primary site, the presence of indeterminate pulmonary nodules, and a worse outcome. Before the current study, ctDNA had only been evaluated in cohorts of patients with metastatic disease of approximately 30 patients or fewer.^13,15^ Here, we show that elevated baseline ctDNA burden among patients with metastatic disease is associated with having a pelvic bone primary site, extrapulmonary metastatic disease, and poor outcomes. We also found that the clinically relevant prognostic thresholds differed between patients with localized and metastatic disease. These studies prospectively validate the prognostic impact of pretreatment ctDNA burden.

We also identified patients with small tumors, low ctDNA, and favorable genomics who have excellent outcomes. The low-risk group studied herein was informed by the validation of pretreatment ctDNA burden and numerous prior studies showing the prognostic impact of *STAG*2 and *TP*53 alterations and tumor size. Among patients with metastatic disease, using metastatic pattern, ctDNA burden, and tumor genomics detected in ctDNA, we identify an intermediate-risk group that is more likely to be cured with standard therapy. Conversely, we also identify a group of patients with *TP*53 mutations who all suffer disease relapse within 2 years of diagnosis. Our approach to improving outcomes for these disparate groups should differ.

Measuring treatment response in bone sarcomas is challenging. Among patients treated with the VIDE regimen, on-therapy ctDNA burden after two cycles of therapy was prognostic.^15^ We show that ctDNA levels drop predictably in response to interval-compressed VDC/IE for patients with localized and metastatic EWS. In our more limited cohort of patients with localized EWS and on-therapy ctDNA, we were unable to detect a difference in outcomes by ctDNA response before cycle 2. Conversely, among patients with metastatic disease, those with residual ctDNA after one cycle of chemotherapy had dismal outcomes. Validation of the prognostic impact of on-treatment ctDNA levels using novel technologies with improved sensitivity will be a focus of future studies.

The LEOPARD study was designed to validate the prognostic impact of baseline ctDNA and was conducted during the COVID-19 pandemic, and therefore, fewer patients with localized EWS had serial samples available. This limited our ability to study on-treatment time points. We were also unable to show that patients with *STAG*2 mutations in our localized cohort had inferior outcomes compared with patients with *STAG*2*^WT^* disease, possibly because of inadequate power or differences in methodology. Our risk groups were based on prespecified validated biomarkers, and prospective implementation of risk stratification needs to be conducted in the context of a clinical trial to fully validate these findings. Finally, the TranSS-Seq assay was designed to allow for detection of relevant EWS fusions without prior knowledge of the specific fusion breakpoints although we acknowledge that other approaches might have greater sensitivity.

In summary, we provide a comprehensive view of ctDNA detection at baseline and over time, secondary mutation detection, and prognostic potential of this biomarker in EWS. We have prospectively validated that ctDNA burden at diagnosis is associated with inferior EFS. We can detect *STAG*2 and *TP*53 mutations in the ctDNA, and these variants are also associated with poor outcome in EWS. These data were generated using a previously validated assay that is currently being developed into a clinical assay. Taken together, ctDNA is now positioned for use as a clinical prognostic factor in future trials for newly diagnosed EWS.

## Supplementary Material

Data Supplement

## Figures and Tables

**FIG 1.**
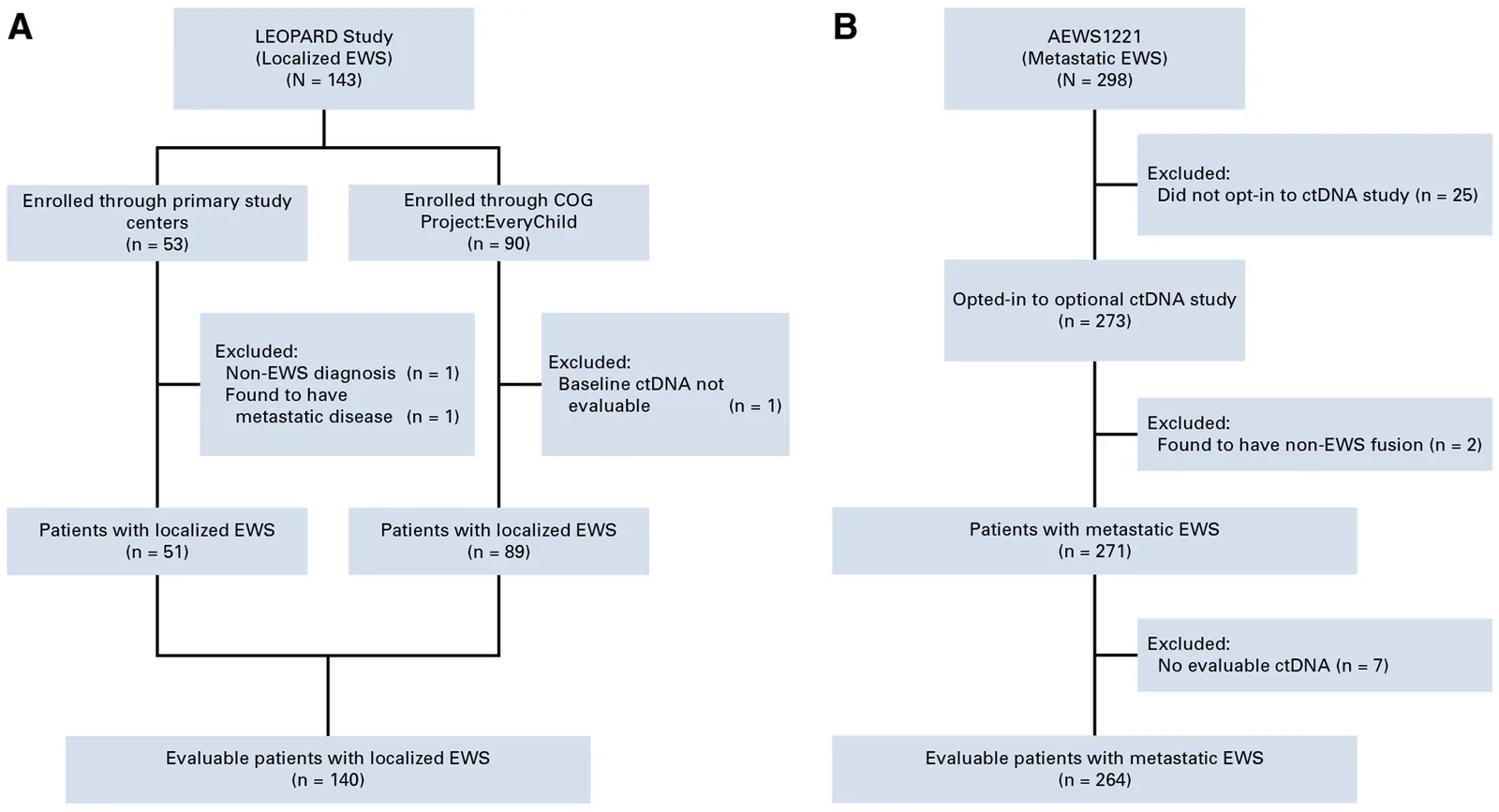
Flow diagram of patients with (A) localized EWS on the LEOPARD study and (B) metastatic EWS on AEWS1221. COG, Children’s Oncology Group; ctDNA, circulating tumor DNA; EWS, Ewing sarcoma.

**FIG 2.**
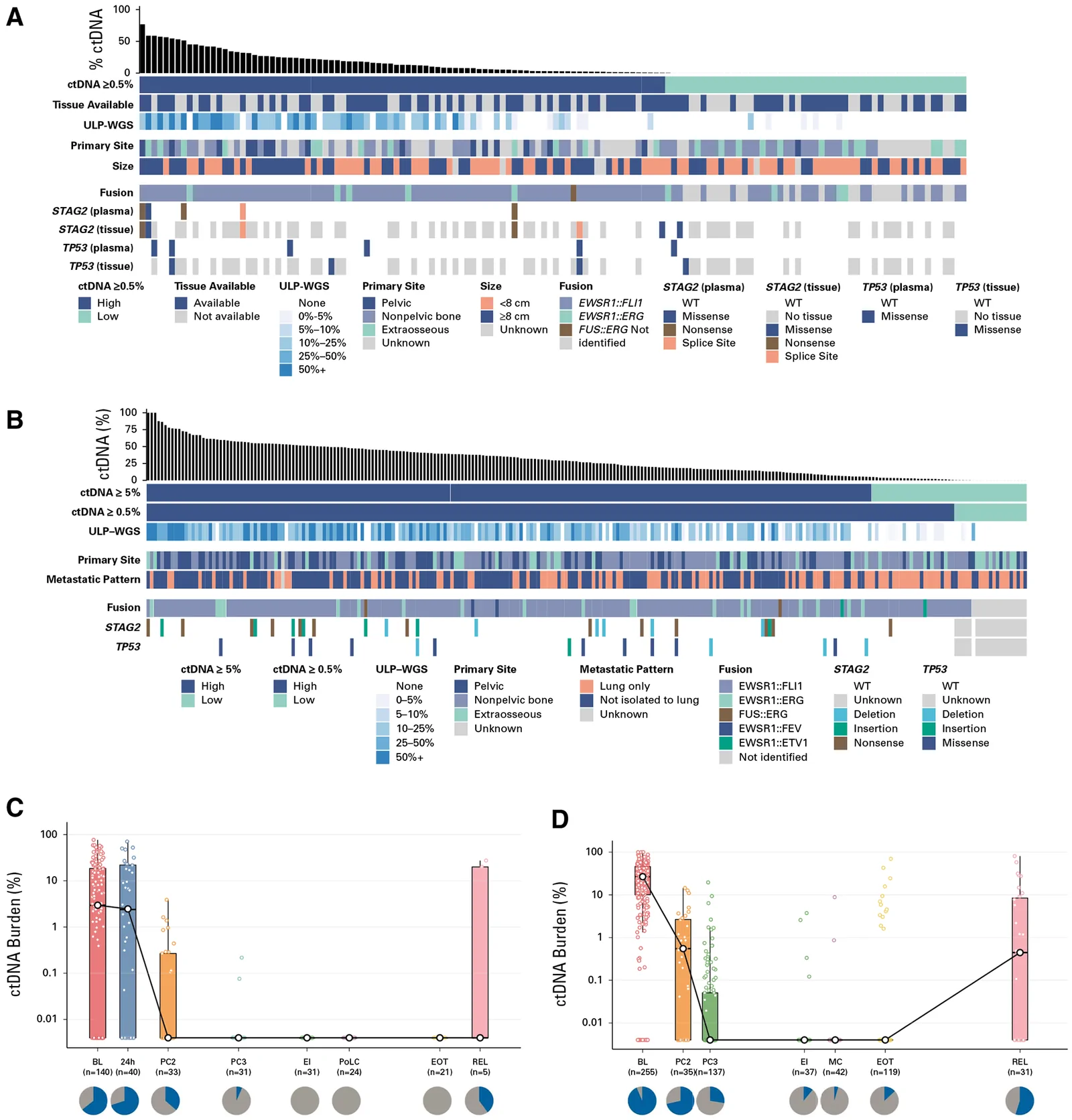
Comprehensive ctDNA profiling and clinical annotation of patients with localized and metastatic EWS. Oncoprints of baseline ctDNA and molecular data for patients with (A) localized EWS and (B) metastatic EWS. Fusion-based serial ctDNA measurements as percent ctDNA burden in a log scale for patients with (C) localized EWS and (D) metastatic EWS. Patients with undetectable ctDNA are plotted as 0.004. n represents the number of patients with evaluable samples at each time point. Pie charts represent the fraction of patients with detectable ctDNA at that time point (blue). Time points represent nominal time points at which samples were drawn and are not shown at an approximated time scale. 24 h, 24-hour; BL, baseline; ctDNA, circulating tumor DNA; EI, end induction; EOT, end-of-consolidation therapy; EWS, Ewing sarcoma; MC, mid consolidation; PC2, Precycle 2; PC3, Precycle 3; PoLC, postlocal control; REL, relapse; ULP-WGS, ultralow-pass whole-genome sequencing; WT, wild type.

**FIG 3.**
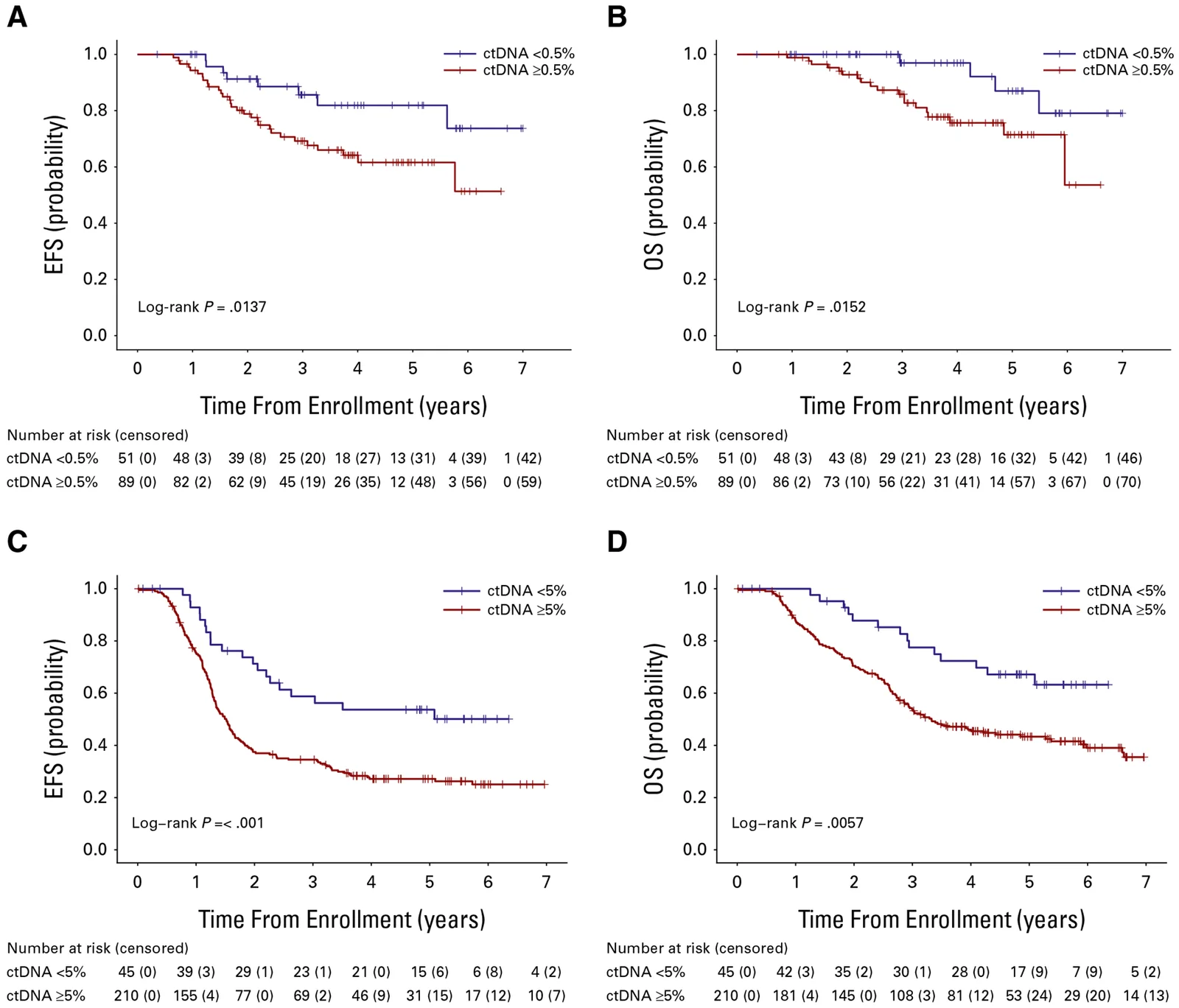
Elevated pretreatment ctDNA burden is associated with inferior EFS. (A) EFS and (B) OS stratified by pretreatment ctDNA burden among patients with localized EWS. (C) EFS and (D) OS stratified by pretreatment ctDNA burden in metastatic EWS. ctDNA, circulating tumor DNA; EFS, event-free survival; EWS, Ewing sarcoma; OS, overall survival.

**FIG 4.**
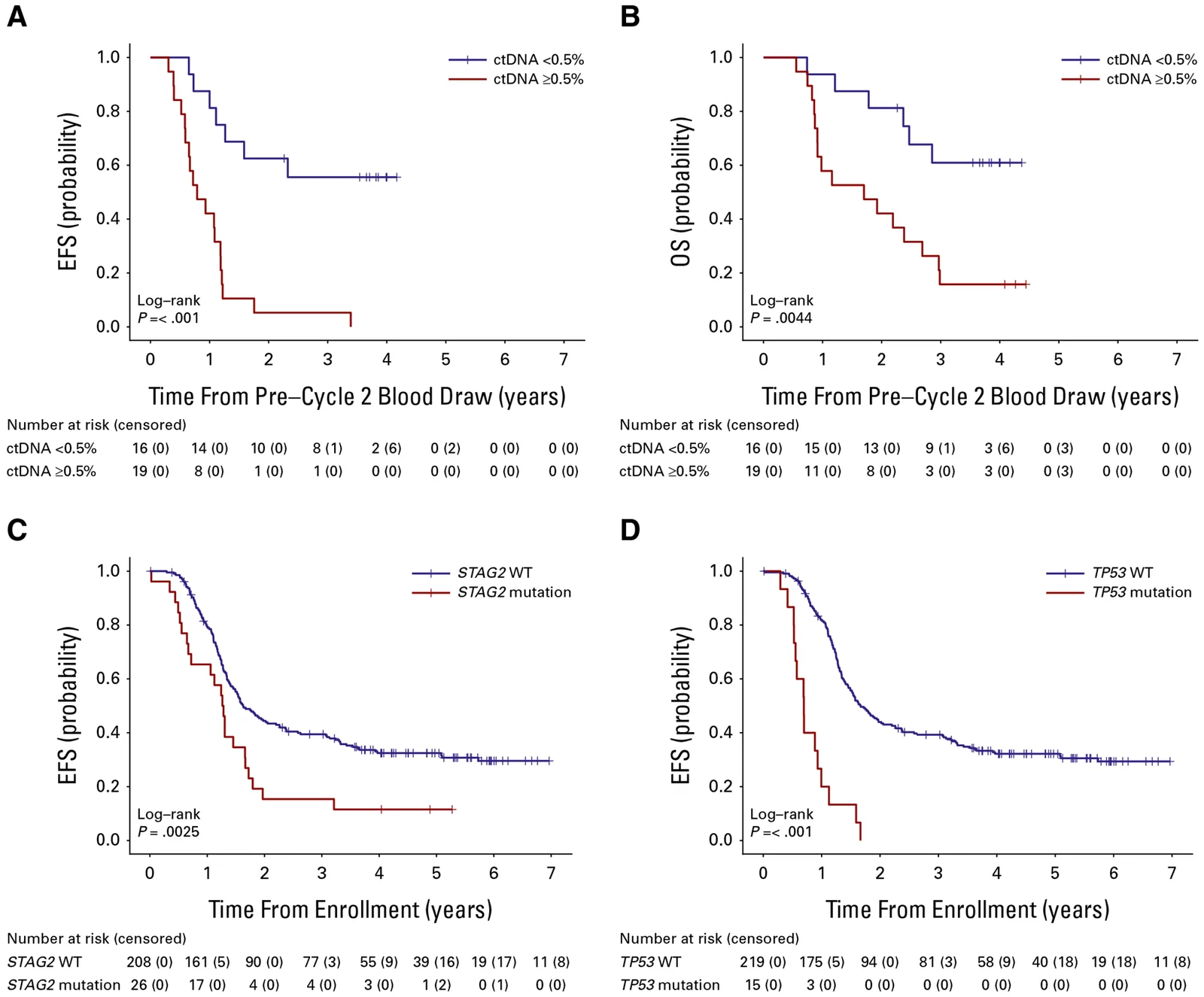
Analysis of on-therapy ctDNA and secondary molecular events in metastatic EWS. The presence of elevated ctDNA burden before cycle 2 is associated with inferior (A) EFS and (B) OS. (C) *STAG2* mutations present in ctDNA at baseline are associated with inferior EFS. (D) *TP53* mutations present in ctDNA at baseline are associated with inferior EFS. ctDNA, circulating tumor DNA; EFS, event-free survival; EWS, Ewing sarcoma; OS, overall survival.

**FIG 5.**
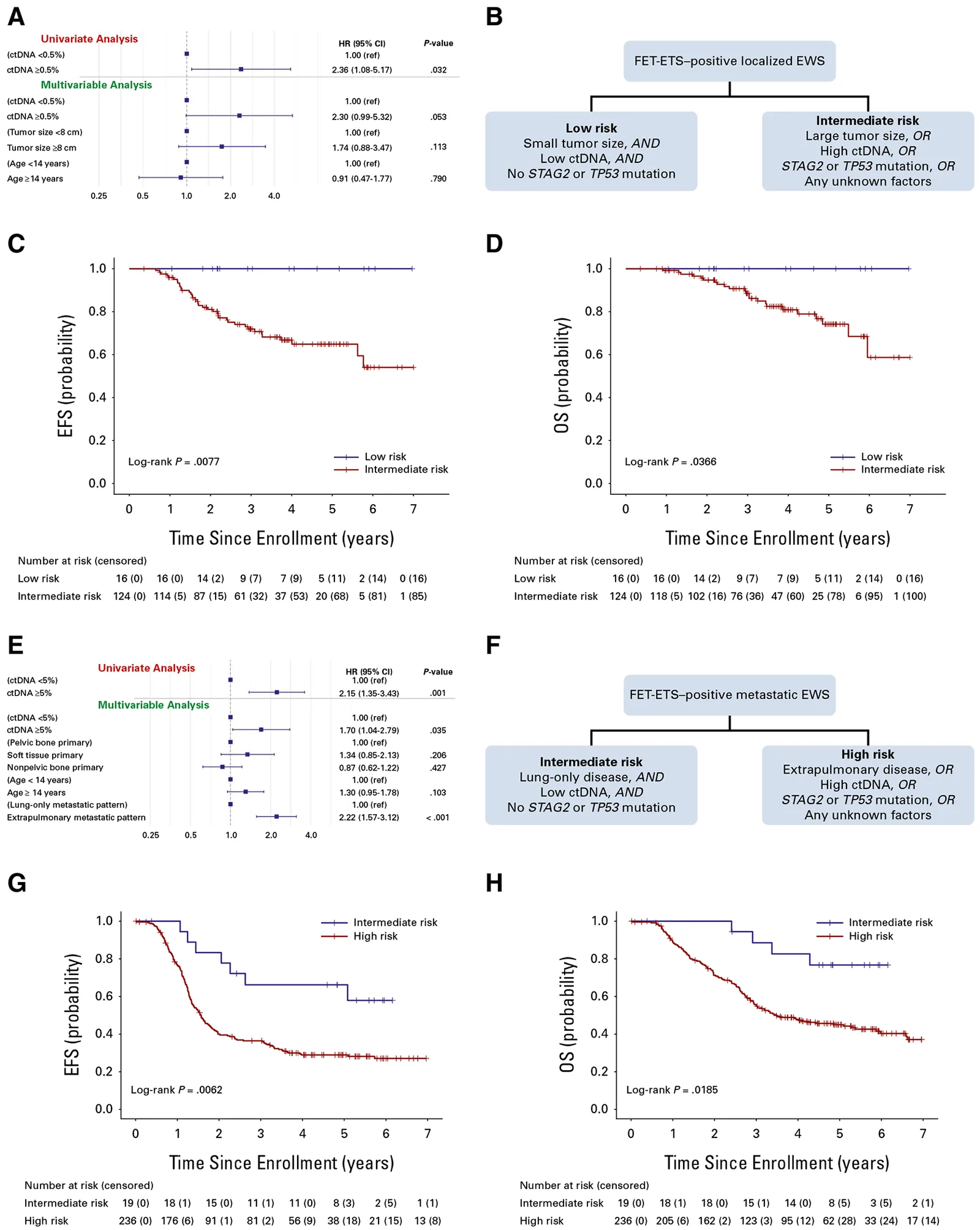
Multivariable analyses show that pretreatment ctDNA burden of ≥0.5% for patients with localized disease and ≥5% for patients with metastatic disease provide independent prognostic value. Integration of clinical features, ctDNA, and molecular features identifies discrete risk groups for patients with EWS. (A) Univariate (n = 140) and multivariable (n = 134) Cox models of EFS for patients with complete data including baseline ctDNA, tumor size, and age. (B) We hypothesized that patients with small tumors, low ctDNA, and no *STAG2* or *TP53* mutations would represent a low-risk group among patients with localized disease that could inform stratification schemas on future trials. We found that compared with patients with large tumors, high ctDNA, *STAG2* or *TP53* mutations, and/or unknown features, patients in this low-risk group had superior (C) EFS and (D) OS. (E) Among patients with metastatic disease, we show the univariate (ctDNA ≥5%; n = 255) and multivariable (n = 255) Cox models of EFS for ctDNA ≥5%, age ≥14 years, primary site and nonpelvic bone primary site and soft tissue primary site (pelvic bone as reference), and extrapulmonary metastatic pattern (lung-only as reference). (F) We hypothesized that patients with lung-only disease, low ctDNA (<5%), and favorable genomics would inform an intermediate-risk group, in contrast to patients with any high-risk feature. We found that these groups had differential (G) EFS and (H) OS. (B) and (F) were created using https://BioRender.com. ctDNA, circulating tumor DNA; EFS, event-free survival; EWS, Ewing sarcoma; OS, overall survival.

**TABLE 1.** Patient Characteristics

Characteristic	LEOPARD Cohort (n = 140)	AEWS1221 Cohort (n = 264)
Age, years, median (range)	14.3 (2.2-35.5)	15.0 (0.0-38.0)
Male sex, No. (%)	91 (65)	148 (56)
Treatment, No. (%)		
VDC/IE	NA	131 (50)
VDC/IE + ganitumab	NA	133 (50)
Race,^a^ No. (%)		
White	116 (86)	214 (93)
Non-White	19 (14)	15 (7)
Ethnicity,^b^ No. (%)		
Hispanic or Latino	17 (13)	41 (17)
Non-Hispanic or Latino	110 (87)	195 (82)
Primary site,^c^ No. (%)		
Nonpelvic bone	77 (56)	137 (52)
Pelvic bone	28 (20)	87 (33)
Extraosseous	32 (23)	40 (15)
Stage, No. (%)		
Localized	140 (100)	0 (0)
Metastatic	0 (0)	264 (100)
Indeterminate pulmonary nodules,^d^ No. (%)		
Present	11 (8)	NA
Absent	126 (92)	NA
Metastatic pattern, No. (%)		
Lung-only	NA	105 (40)
Extrapulmonary	NA	159 (60)
Tumor size,^e^ No. (%)		
<8 cm	68 (51)	NA
≥8 cm	66 (49)	NA

Abbreviations: IE, ifosfamide/etoposide; VDC, vincristine/doxorubicin/cyclophosphamide.

a Race was available for 135 patients in the LEOPARD study and 229 patients in AEWS1221.

b Ethnicity was available for 127 patients in the LEOPARD study and 236 patients in AEWS1221.

c Primary site was available for 137 patients in the LEOPARD study.

d Indeterminate pulmonary nodules were defined as pulmonary nodules that did not meet institutional criteria for metastatic disease and were available for 137 patients in LEOPARD.

e Tumor size was available for 134 patients in the LEOPARD study. Tumor size was not available for patients treated in AEWS1221.

**TABLE 2.** Associations Between Clinical Features and ctDNA Burden

All Patients		
Stage	Median ctDNA Burden (range)	*P*

Localized (n = 140)	3.0 (UD, 76.8)	<.001

Metastatic (n = 255)	26.6 (UD, 100)	
LEOPARD (localized EWS, n = 140)		

Characteristic	Median ctDNA burden (range)	*P*

Age, years		

≥14	4.6 (UD, 58.8)	.408

<14	2.3 (UD, 76.8)	

Race		

White	2.6 (UD, 76.8)	.180

Non-White	18.1 (UD, 45.1)	

Ethnicity		

Non-Hispanic or Latino	2.2 (UD, 54.5)	.951

Hispanic or Latino	3.0 (UD, 58.9)	

Size of primary tumor, cm, median (%)		

<8	1.1 (UD, 56.4)	.032

≥8	6.2 (UD, 76.8)	

Primary site, median (%)		

Nonpelvic bone	2.2 (UD, 58.8)	.001^a^

Pelvic bone	10.6 (UD, 76.8)	

Extraosseous	0.3 (UD, 58.9)	

Indeterminate pulmonary nodules, median (%)		

Present	28.5 (UD, 58.9)	.018

Absent	2.8 (UD, 76.8)	
AEWS1221 (metastatic EWS, n = 255)		

Characteristic	Median ctDNA burden (range)	*P*

Age, years		

≥14	34.5 (UD, 100)	.002

<14	19.7 (UD, 76.8)	

Race		

White	28.3 (UD, 100)	.594

Non-White	20.5 (UD, 87.5)	

Ethnicity		

Non-Hispanic or Latino	28.3 (UD, 100)	.248

Hispanic or Latino	20.2 (UD, 100)	

Primary site, median (%)		

Nonpelvic bone	21.0 (UD, 100)	<.001^a^

Pelvic bone	38.8 (UD, 87.5)	

Extraosseous	15.7 (UD, 100)	

Metastatic pattern, median (%)		

Lung only	16.6 (UD, 100)	<.001

Not lung only	35.9 (UD, 100)	

Abbreviations: ctDNA, circulating tumor DNA; EWS, Ewing sarcoma; UD, undetectable.

a Pelvic bone versus other.

## Data Availability

Deidentified clinical data supporting the findings of this study are available from the corresponding authors upon reasonable request, subject to approval by the Children’s Oncology Group and institutional review boards. The LEOPARD study is registered at ClinicalTrials.gov (identifier: NCT06068075). AEWS1221 is registered at ClinicalTrials.gov (identifier: NCT02306161).

## CONTEXT

### Key Objective

To prospectively validate pretreatment circulating tumor DNA (ctDNA) burden as a prognostic biomarker and propose clinical-molecular risk groups in patients with localized and metastatic Ewing sarcoma (EWS).

### Knowledge Generated

Pretreatment ctDNA burden is a validated prognostic factor in EWS, and integration of ctDNA, secondary genomic events, and clinical features identifies novel low-risk groups.

### Relevance (*S. Bhatia*)

This study identifies the importance of pre-treatment ctDNA as an integral biomarker in risk stratification of patients with EWS in future frontline trials.*

*Relevance section written by *JCO* Associate Editor Smita Bhatia, MD, MPH, FASCO.

## References

[1] Riggi N, Suva ML, Stamenkovic I: Ewing’s sarcoma. N Engl J Med 384:154–164, 2021 10.1056/NEJMra2028910 33497548

[2] Leavey PJ, Laack NN, Krailo MD, : Phase III trial adding vincristine-topotecan-cyclophosphamide to the initial treatment of patients with nonmetastatic Ewing sarcoma: A Children’s Oncology Group report. J Clin Oncol 39:4029–4038, 2021 10.1200/JCO.21.00358 PMC8677904 34652968

[3] Womer RB, West DC, Krailo MD, : Randomized controlled trial of interval-compressed chemotherapy for the treatment of localized ewing sarcoma: A report from the Children’s Oncology Group. J Clin Oncol 30:4148–4154, 2012 10.1200/JCO.2011.41.5703 PMC3494838 23091096

[4] Brennan B, Kirton L, Marec-Berard P, : Comparison of two chemotherapy regimens in patients with newly diagnosed Ewing sarcoma (EE2012): An open-label, randomised, phase 3 trial. Lancet 400:1513–1521, 2022 10.1016/S0140-6736(22)01790-1 36522207

[5] Marina NM, Liu Q, Donaldson SS, : Longitudinal follow-up of adult survivors of Ewing sarcoma: A report from the Childhood Cancer Survivor Study. Cancer 123:2551–2560, 2017 10.1002/cncr.30627 PMC5474122 28222219

[6] Dieffenbach BV, Murphy AJ, Liu Q, : Cumulative burden of late, major surgical intervention in survivors of childhood cancer: A report from the Childhood Cancer Survivor Study (CCSS) cohort. Lancet Oncol 24:691–700, 2023 10.1016/S1470-2045(23)00154-7 PMC10348667 37182536

[7] Crompton BD, Stewart C, Taylor-Weiner A, : The genomic landscape of pediatric Ewing sarcoma. Cancer Discov 4:1326–1341, 2014 10.1158/2159-8290.CD-13-1037 25186949

[8] Tirode F, Surdez D, Ma X, : Genomic landscape of Ewing sarcoma defines an aggressive subtype with co-association of STAG2 and TP53 mutations. Cancer Discov 4:1342–1353, 2014 10.1158/2159-8290.CD-14-0622 PMC4264969 25223734

[9] Brohl AS, Solomon DA, Chang W, : The genomic landscape of the Ewing sarcoma family of tumors reveals recurrent STAG2 mutation. PLoS Genet 10:e1004475, 2014 10.1371/journal.pgen.1004475 PMC4091782 25010205

[10] Adane B, Alexe G, Seong BKA, : STAG2 loss rewires oncogenic and developmental programs to promote metastasis in Ewing sarcoma. Cancer Cell 39:827–844.e10, 2021 10.1016/j.ccell.2021.05.007 PMC8378827 34129824

[11] Surdez D, Zaidi S, Grossetête S, : STAG2 mutations alter CTCF-anchored loop extrusion, reduce cis-regulatory interactions and EWSR1-FLI1 activity in Ewing sarcoma. Cancer Cell 39:810–826.e9, 2021 10.1016/j.ccell.2021.04.001 33930311

[12] Gillani R, Shulman DS, DelRocco NJ, : Molecular characterization informs prognosis in patients with localized Ewing sarcoma: A report from the Children’s Oncology Group. J Clin Oncol 43:3750–3759, 2025 10.1200/JCO-25-00157 PMC12614438 41183255

[13] Shulman DS, Klega K, Imamovic-Tuco A, : Detection of circulating tumour DNA is associated with inferior outcomes in Ewing sarcoma and osteosarcoma: A report from the Children’s Oncology Group. Br J Cancer 119:615–621, 2018 10.1038/s41416-018-0212-9 PMC6162271 30131550

[14] Krumbholz M, Hellberg J, Steif B, : Genomic EWSR1 fusion sequence as highly sensitive and dynamic plasma tumor marker in ewing sarcoma. Clin Cancer Res 22:4356–4365, 2016 10.1158/1078-0432.CCR-15-3028 27283964

[15] Krumbholz M, Eiblwieser J, Ranft A, : Quantification of translocation-specific ctDNA provides an integrating parameter for early assessment of treatment response and risk stratification in Ewing sarcoma. Clin Cancer Res 27:5922–5930, 2021 10.1158/1078-0432.CCR-21-1324 34426444

[16] DuBois SG, Krailo MD, Glade-Bender J, : Randomized phase III trial of ganitumab with interval-compressed chemotherapy for patients with newly diagnosed metastatic Ewing sarcoma: A report from the Children’s Oncology Group. J Clin Oncol 41:2098–2107, 2023 10.1200/JCO.22.01815 PMC10082251 36669140

[17] Shulman DS, Merriam P, Choy E, : Phase 2 trial of palbociclib and ganitumab in patients with relapsed Ewing sarcoma. Cancer Med 12:15207–15216, 2023 10.1002/cam4.6208 PMC10417097 37306107

[18] Greenwood M: A Report on the Natural Duration of Cancer, Volume 33. Reports on Public Health and Medical Subjects. Ministry of Health, 1926

[19] Shulman DS, Chen S, Hall D, : Adverse prognostic impact of the loss of STAG2 protein expression in patients with newly diagnosed localised Ewing sarcoma: A report from the Children’s Oncology Group. Br J Cancer 127:2220–2226, 2022 10.1038/s41416-022-01977-2 PMC9726932 36221002

