# Supplementary material for: Circulating Tumor DNA Profiling Defines Risk Classification in Patients With Ewing Sarcoma: A Report From the Children’s Oncology Group and the LEOPARD Study
Source: J Clin Oncol. Author manuscript; Available in PMC 2026 Nov 13. (PMC13766215; doi:10.1200/JCO-26-00285)
Supplement: Supplementary file 1 [file NIHMS2219624-supplement-Data_Supplement.pdf]

Appendix Methods

Categorical variables were reported as frequencies and percentages stratified by subgroup. Continuous variables were described using median and range. To test for associations between ctDNA-defined subgroups versus clinical measures, chi-squared and Fisher's exact tests were used for categorical variables, and a Wilcoxon rank-sum test was used for continuous variables.

Circulating Tumor DNA and Tissue Analysis

In brief, blood samples were collected in cell stabilizing blood collection tubes at set timepoints prior to and on treatment. Double-spun plasma was stored at -80°C until cell-free DNA was extracted and sequencing libraries were prepared. Tumor DNA was extracted from FFPE tumor tissue using Covaris truXTRAC FFPE total NA or Qiagen AllPrep DNA/RNA FFPE kits. Illumina sequencing libraries were prepared for both cell-free DNA and FFPE tumor DNA.

For Ultra-Low Passage Whole Genome Sequencing (ULP-WGS), DNA libraries were sequenced on Illumina sequencers to a target of >0.1x coverage. Analysis was performed using the IchorCNA analysis pipeline with manual curation.[1] This methodology quantifies ctDNA based on copy number changes across the genome and has been previously validated in pediatric solid tumors.[2]

For fusion identification and quantitation, an updated version of the previously described custom capture assay TranSS-Seq[2] was used. This assay uses baits for intronic regions of genes likely to be involved in Ewing Sarcoma and Ewing-like diseases. The updated version uses biotinylated synthetic DNA oligonucleotide probes from Twist Bioscience and targets relevant intronic regions of the following genes for translocation detection: *EWSR1*, *FUS*, *CIC*, *CCNB3*, *PAX3*, *PAX7*, *BCOR*, *DUX4L6*, *DUX4L7*, *ERG*, *FLI1*, and *FOXO1*. The updated version was validated using the same samples used to validate the prior version. Two separate fusion

detection algorithms were used to identify translocation breakpoints, SvABA[3] and BreaKmer.[4] PSTQuant, a previously described custom algorithm,[2] was then used to quantify the amount of circulating tumor DNA in the sample by comparing translocated and wild-type reads at the fusion breakpoint.

TranSS-Seq also includes baits for exonic regions of the genes *TP53* and *STAG2*. Mutations in these genes were identified through use of the algorithm mutect2, a part of the Broad Institute's Genome Analysis ToolKit (GATK , RRID:SCR_001876) (https://gatk.broadinstitute.org/hc/en-us/articles/360037593851-Mutect2).

Independent variables

Among patients with localized EWS, the primary independent variable was ctDNA detected at baseline, defined as ≥0.5% or <0.5% ctDNA, based on data from our prior study and improved assay sensitivity.[14] We evaluated 3% as a secondary pre-planned cutpoint as an approximation of the median baseline ctDNA burden among patients with localized EWS. Among patients with metastatic EWS, for whom the ctDNA detection rate was significantly higher at baseline with a wide dynamic range, we evaluated 0.5% and 5% ctDNA burden. ctDNA burden as a continuous variable was evaluated in both cohorts. The presence of *STAG2* and *TP53* alterations in baseline samples were secondary independent variables.

We collected key clinical covariates at baseline. Age was dichotomized as ≥ or <14 years at enrollment. For the localized cohort, tumor size was dichotomized as ≥ or <8 cm. Primary tumor size was not a collected data element on AEWS1221.

Dependent variables

The primary endpoint was EFS, defined as time from ctDNA specimen collection to first event (disease progression, second malignancy, or death from any cause). EFS was the pre-specified primary endpoint of the LEOPARD study to align with the endpoint used in our prior analysis and the standard endpoint used in most frontline bone sarcoma clinical trials.[2,3,14] Patients without an event were censored at last follow-up. Overall survival (OS) was defined as time from ctDNA specimen collection to death from any cause, with alive patients censored at the time of last follow-up. Additional secondary analyses were performed using EFS and OS time landmarked starting from the date of the relevant subsequent ctDNA blood draw.

1. Adalsteinsson VA, Ha G, Freeman SS, et al: Scalable whole-exome sequencing of cell-free DNA reveals high concordance with metastatic tumors. Nat Commun 8:1324, 2017

2. Klega K, Imamovic-Tuco A, Ha G, et al: Detection of Somatic Structural Variants Enables Quantification and Characterization of Circulating Tumor DNA in Children With Solid Tumors [Internet]. JCO Precis Oncol 2018:1–13, 2018Available from: https://www.ncbi.nlm.nih.gov/pubmed/30027144

3. Wala JA, Bandopadhayay P, Greenwald NF, et al: SvABA: genome-wide detection of structural variants and indels by local assembly. Genome Res 28:581–591, 2018

4. Abo RP, Ducar M, Garcia EP, et al: BreaKmer: detection of structural variation in targeted massively parallel sequencing data using kmers. Nucleic Acids Res 43:e19–e19, 2015

Supplementary Table: Comparison of clinical and demographic features between ProjectEveryChild (n=89) and LEOPARD (n=51)

**Characteristic**	**ProjectEveryChild** (n = 89)	**LEOPARD** (n = 51)	**p-value**
Age (years), median (range)	13.9 (4.5, 23.2)	14.9 (2.2, 35.5)	0.246
Sex			0.672
Female, n (%)	30 (34)	19 (37)	
Male, n (%)	59 (66)	32 (63)	
Race			0.014
Non-white, n (%)	7 (8)	12 (24)	
White, n (%)	77 (92)	39 (76)	
Unknown	5	0	
Ethnicity			0.952
Hispanic or Latino, n (%)	11 (13)	6 (14)	
Non-Hispanic or Latino, n (%)	72 (87)	38 (86)	
Unknown	6	7	
Indeterminate pulmonary nodules			0.211
Present, n (%)	9 (10)	2 (4)	
Absent, n (%)	77 (90)	49 (96)	
Unknown	3	0	
Primary site			0.533*
non-pelvic bone, n (%)	49 (57)	28 (55)	
pelvic bone, n (%)	19 (22)	9 (18)	
extraosseous, n (%)	18 (21)	14 (27)	

**Characteristic**	**ProjectEveryChild** (n = 89)	**LEOPARD** (n = 51)	**p-value**
Unknown	3	0	
Tumor size			0.261
< 8 cm, n (%)	40 (47)	28 (57)	
≥ 8 cm, n (%)	45 (53)	21 (43)	
Unknown	4	2	

* Pelvic bone vs other

Continuous variables were compared using the Wilcoxon rank-sum test, and categorical variables were compared using the Chi-square test or Fisher's exact test, as appropriate.

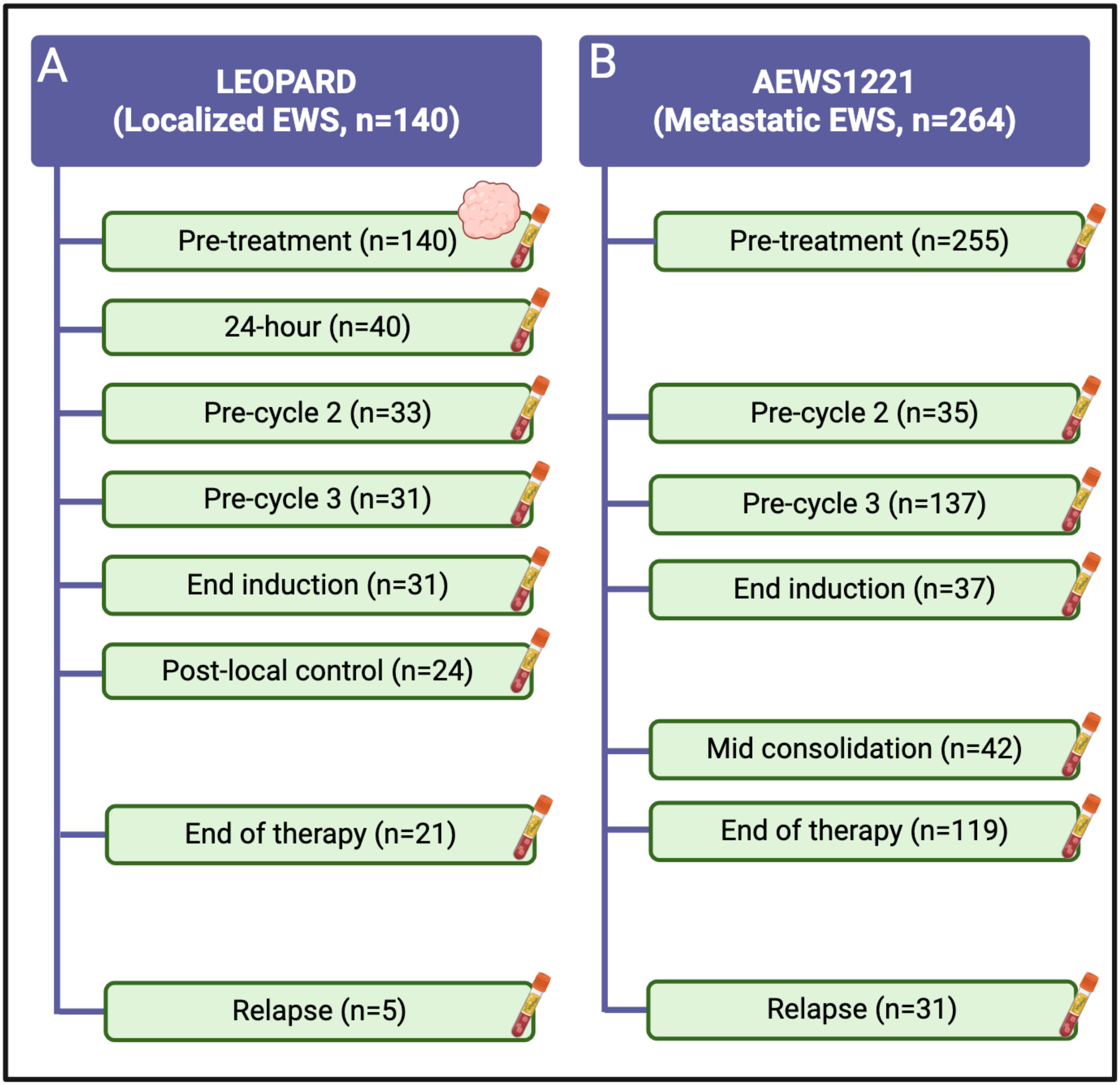

Appendix Fig S1: ctDNA collection schema and collected evaluable samples for patients with localized **(A)** and metastatic EWS **(B)**. Created in https://BioRender.com.

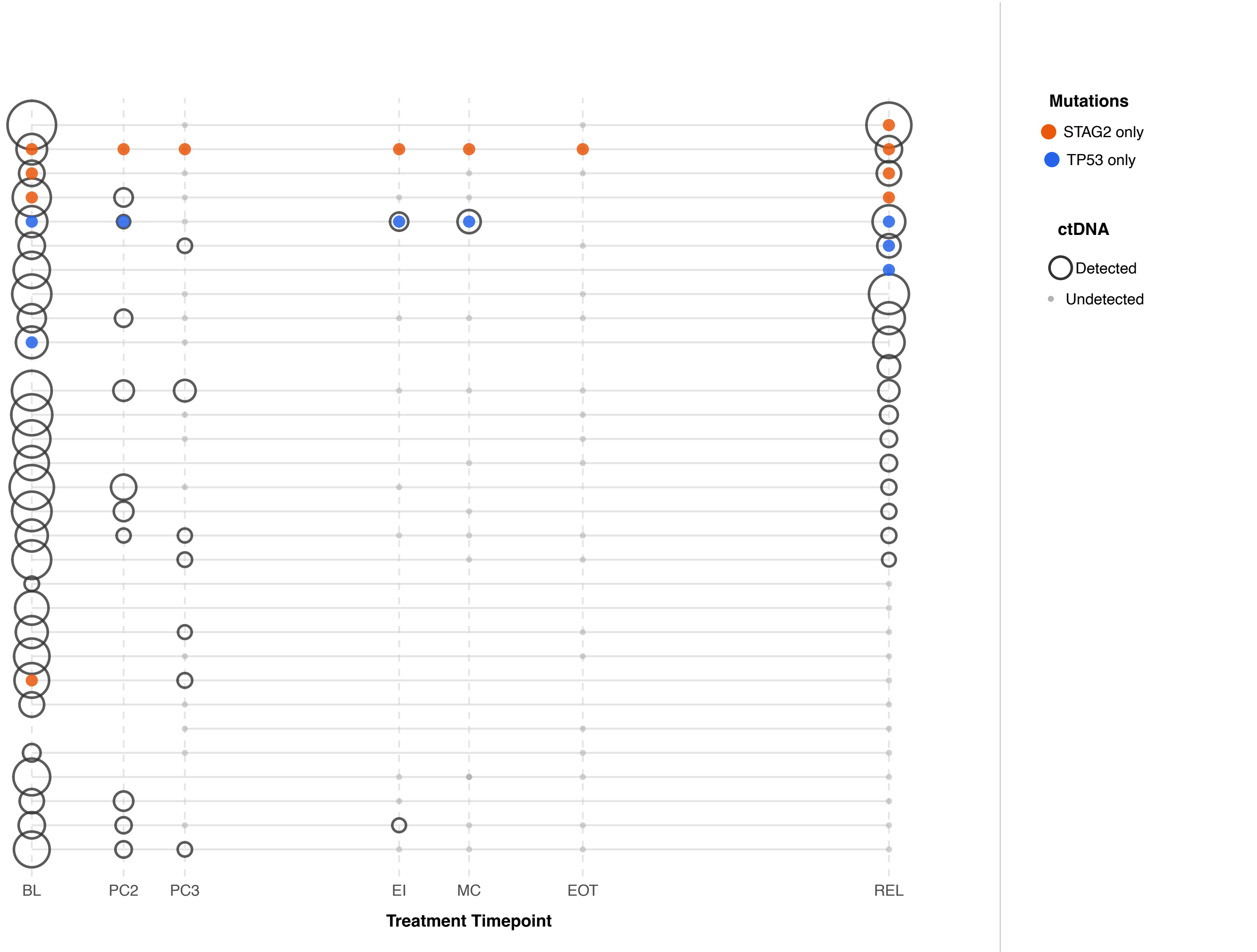

Appendix Fig S2: ctDNA burden over time (black circles) and STAG2 (blue) and TP53 (orange) allelic fraction among 31 patients with available relapse samples. BL=baseline; PC2=Pre-cycle 2; PC3=Pre-cycle 3; EI=End induction; MC=Mid consolidation; REL=Relapse.

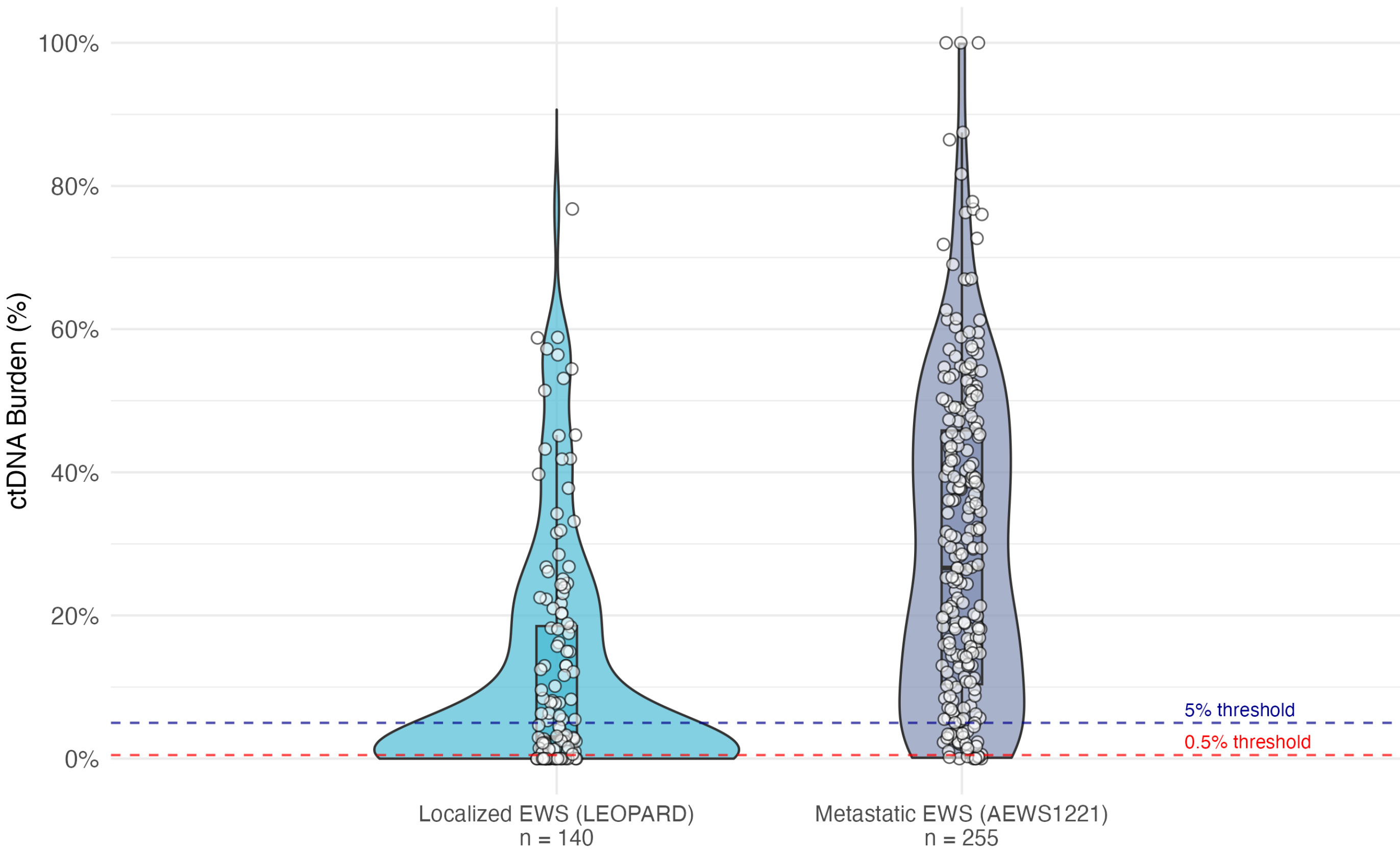

Appendix Fig S3: Patients with localized EWS from the LEOPARD study (left) have lower baseline ctDNA when compared to patients with metastatic EWS from AEWS1221 (right; $p<0.001$).

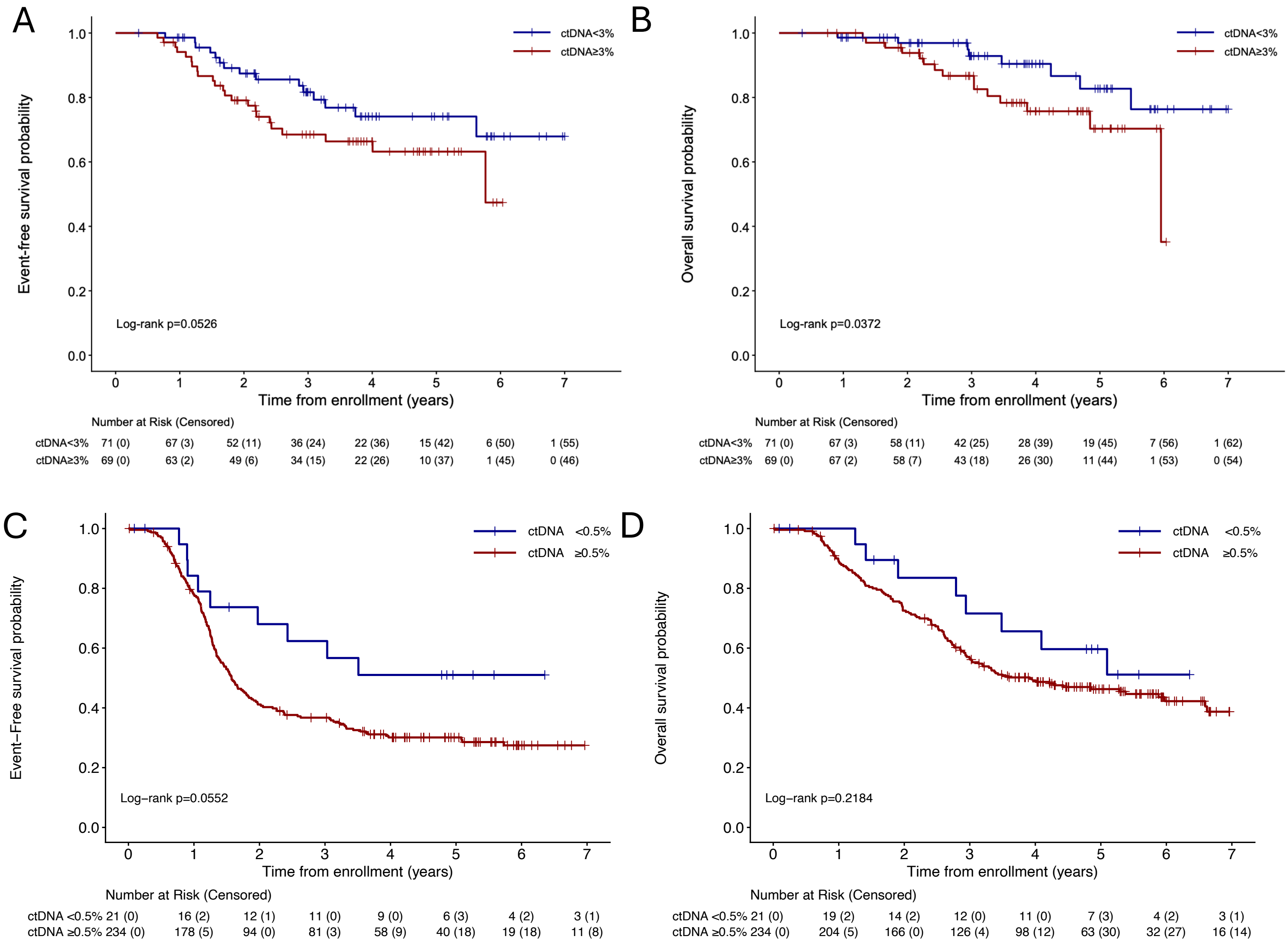

Appendix Fig S4: Survival analyses by secondary ctDNA burden cutpoints
Pre-treatment ctDNA ≥3% is associated with an inferior EFS **(A)** and OS **(B)** among patients with localized EWS. Pre-treatment ctDNA burden ≥0.5% is associated with a nominally worse EFS **(C)** and OS **(D)** among patients with metastatic EWS.

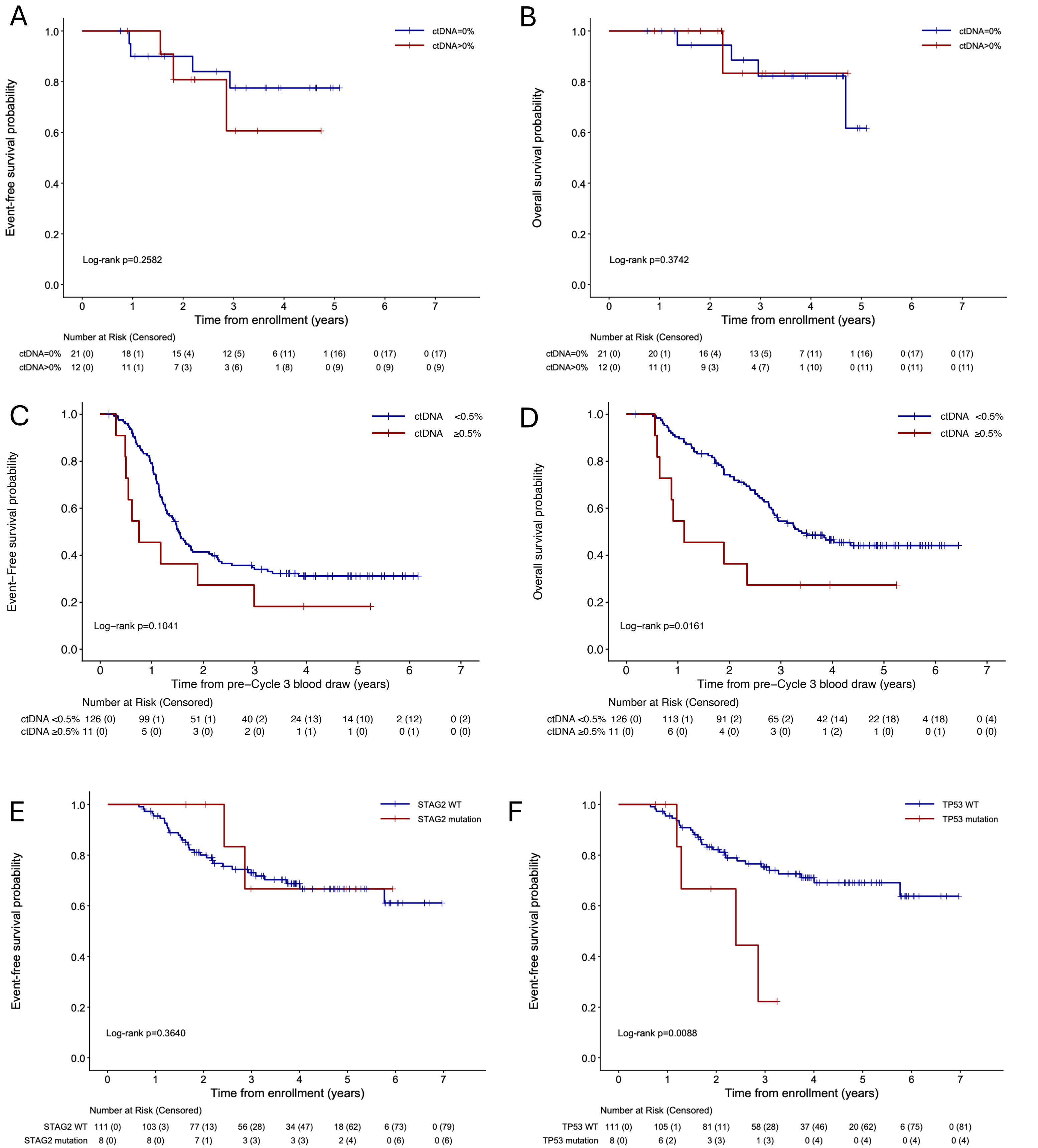

Appendix Fig S5: Survival analyses of on-treatment ctDNA burden and secondary genomic events.
Among patients with localized EWS, detectable ctDNA burden prior to cycle 2 was not associated with EFS **(A)** or OS **(B)**. Among patients with metastatic EWS, ctDNA burden ≥0.5% prior to cycle 3 among patients with metastatic EWS was not associated with EFS **(C)** or OS **(D)**. Among patients with localized EWS, detection of STAG2 mutations in baseline ctDNA or baseline tissue was not associated with a worse EFS **(E)**. Conversely, detection of TP53 mutations in this group of patients was associated with an inferior EFS compared to patients with $TP53^{WT}$ disease **(F)**.

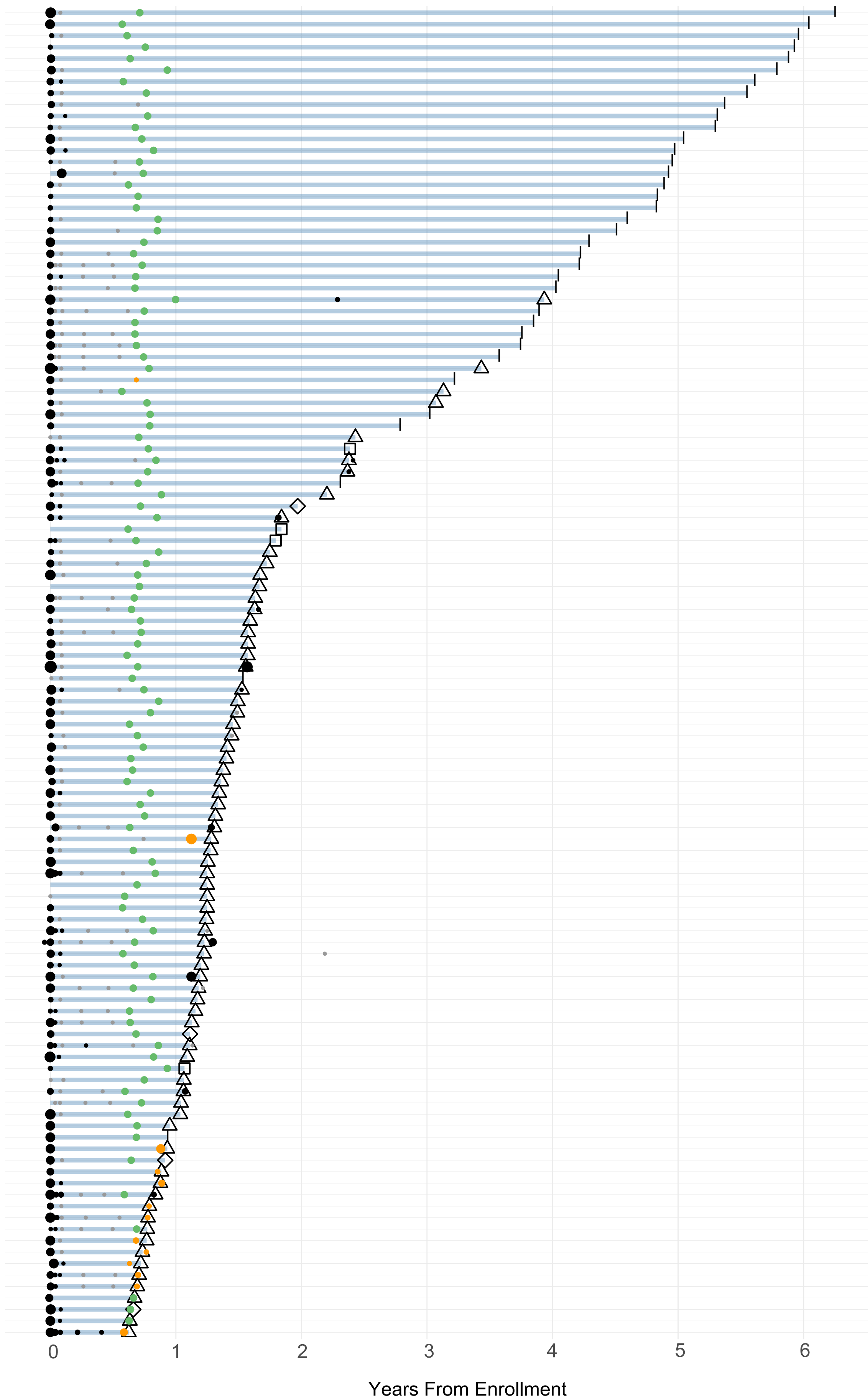

Appendix Fig S6: Swimmer plot of patients with metastatic EWS treated on AEWS1221 with available end-of-therapy ctDNA samples (n=119). Black circle=ctDNA detectable scaled by ctDNA burden; Green circle = undetectable end-of-therapy ctDNA; Orange circle = detectable end-of-therapy ctDNA scaled by ctDNA burden; Triangle = relapse/progression; Square = second malignant neoplasm; Diamond=death as first event.

The REMARK checklist

Item to be reported		Page no.
**INTRODUCTION**		
1	State the marker examined, the study objectives, and any pre-specified hypotheses.	Page 9
**MATERIALS AND METHODS**		
Patients		
2	Describe the characteristics (e.g., disease stage or co-morbidities) of the study patients, including their source and inclusion and exclusion criteria.	
3	Describe treatments received and how chosen (e.g., randomized or rule-based).	Page 9
Specimen characteristics		
4	Describe type of biological material used (including control samples) and methods of preservation and storage.	Page 9
Assay methods		
5	Specify the assay method used and provide (or reference) a detailed protocol, including specific reagents or kits used, quality control procedures, reproducibility assessments, quantitation methods, and scoring and reporting protocols. Specify whether and how assays were performed blinded to the study endpoint.	Page 9
Study design		
6	State the method of case selection, including whether prospective or retrospective and whether stratification or matching (e.g., by stage of disease or age) was used. Specify the time period from which cases were taken, the end of the follow-up period, and the median follow-up time.	Page 8
7	Precisely define all clinical endpoints examined.	
8	List all candidate variables initially examined or considered for inclusion in models.	
9	Give rationale for sample size; if the study was designed to detect a specified effect size, give the target power and effect size.	
Statistical analysis methods		
10	Specify all statistical methods, including details of any variable selection procedures and other model-building issues, how model assumptions were verified, and how missing data were handled.	Page 9
11	Clarify how marker values were handled in the analyses; if relevant, describe methods used for cutpoint determination.	
**RESULTS**		
Data		
12	Describe the flow of patients through the study, including the number of patients included in each stage of the analysis (a diagram may be helpful) and reasons for dropout. Specifically, both overall and for each subgroup extensively examined report the numbers of patients and the number of events.	Page 10-15
13	Report distributions of basic demographic characteristics (at least age and sex), standard (disease-specific) prognostic variables, and tumor marker, including numbers of missing values.	
Analysis and presentation		
14	Show the relation of the marker to standard prognostic variables.	Page 10-15
15	Present univariable analyses showing the relation between the marker and outcome, with the estimated effect (e.g., hazard ratio and survival probability). Preferably provide similar analyses for all other variables being analyzed. For the effect of a tumor marker on a time-to-event outcome, a Kaplan-Meier plot is recommended.	
16	For key multivariable analyses, report estimated effects (e.g., hazard ratio) with confidence intervals for the marker and, at least for the final model, all other variables in the model.	
17	Among reported results, provide estimated effects with confidence intervals from an analysis in which the marker and standard prognostic variables are included, regardless of their statistical significance.	
18	If done, report results of further investigations, such as checking assumptions, sensitivity analyses, and internal validation.	
**DISCUSSION**		
19	Interpret the results in the context of the pre-specified hypotheses and other relevant studies; include a discussion of limitations of the study.	Page 16-17
20	Discuss implications for future research and clinical value.	

Source: McShane LM, Altman DG, Sauerbrei W, Taube SE, Gion M, Clark GM: Reporting recommendations for tumor marker prognostic studies (REMARK). *J Natl Cancer Inst* 2005; 97: 1180-1184.
